# Cancer risk among patients with multiple sclerosis: A cohort study in Isfahan, Iran

**DOI:** 10.22088/cjim.8.3.172

**Published:** 2017

**Authors:** Masoud Etemadifar, Hamidreza Jahanbani-Ardakani, Sara Ghaffari, Maboobeh Fereidan-Esfahani, Hossein Changaei, Nazila Aghadoost, Ameneh Jahanbani Ardakani, Negin Moradkhani

**Affiliations:** 1 Department of Neurology, School of Medicine, Isfahan University of Medical Sciences, Isfahan, Iran; 2 Isfahan Medical Students Research Center (IMRC), Isfahan University of Medical Sciences, Isfahan, Iran.; 3 Medical student, School of Medicine, Isfahan University of Medical Sciences, Isfahan, Iran; 4 School of Basic Sciences, Shahid Madani University of Azarbaijan, Tabriz, Iran; 5 School of Medicine, Kashan University of Medical Sciences, Kashan, Iran; 6 Department of Medical Surgical Nursing (M.Sc. Student), School of Nursing and Midwifery, Iran University of Medical Sciences, Tehran, Iran

**Keywords:** Cancer, Multiple Sclerosis, Cohort, Isfahan, Iran

## Abstract

**Background:**

Multiple sclerosis (MS), a central nervous system (CNS) autoimmune disorder, affects 2.3 million people around the world. Cancer kills around 7.5 million people annually. Both diseases have similar risks and intertwining molecular causes. Most studies focusing on MS and cancer have found an insignificant difference or reduction in the amount of cancer found in the MS community.

**Methods:**

We performed a cohort study using data from Isfahan Multiple Sclerosis Society (IMSS) and Isfahan cancer society and followed-up for 8 years on average (2006-2014). All of the 1718 MS patients were diagnosed according to McDonald’s criteria, then standardized incidence ratio and the numbers of expected cancer case were calculated.

**Results:**

While patients had an insignificant change in cancer prevalence, men had fewer cancer cases and women showed an increased prevalence of cancer. Certain types of cancer proved statistically significant. Breast cancer, nervous system cancers, and lymphoma were elevated in the cohort.

**Conclusion:**

Our results support the hypothesis that MS significantly affects certain cancers in a protective or associative manner. All cancer rates, except breast cancer, cancers located in the nervous system, and lymphomas were reduced in cohort, suggesting that unregulated immune function may provide protective effects to MS patients against cancer.

Multiple sclerosis (MS) is a debilitating autoimmune disease of the central nervous system (CNS) that causes demyelination of nerve fibers in the brain and spinal cord. While patients experience different levels of symptoms and patterns of affliction, the disease often results in increasingly severe levels of disability. It is still unknown what causes MS, though certain risk factors such as genetic predisposition, lifestyle, environment, and previous infection have been identified (1). While so much remains unknown about the root causes of MS, even more is unknown about how MS interacts with other clinical presentations of the disease. As the global medical community seeks a deeper understanding of this debilitating disease, there has been a rising interest to discover if MS has any definitive interactions with other diseases. Cancer has many of the same risk factors as MS (2). Similarly, as in MS, it is still not understood how so many risk factors integrate to cause cancer pathogenesis. Determining if cancer occurs at a higher, lower, or average rate in MS patients compared to the general population could suggest further directions for both MS and cancer research. There have been many epidemiological studies indicating that MS may have a protective role with regard to cancer (3-8).

In other words, the risk amongst MS patients for cancer was lower than in the general population. Fewer studies have found an increased risk of cancer overall for people living with MS. Under certain circumstances where the prevalence of cancer increased in the cohort, the difference could be attributed to an increased amount of medical surveillance in patients (9). However, the notion that MS is protective against cancer is hardly a fact. Data from the National Health Insurance System of Taiwan showed an increased risk of cancer overall and an increased risk for breast cancer alone (10). There have also been results that suggest cancer incidence occurs at the same rate in MS cohorts as it does in the general population (11, 12). 

To the best of our knowledge, there is no cohort study to investigate the risk and prevalence of cancers among patients with multiple sclerosis in Iran. In this study, patients with an average of eight years were followed-up and the prevalence and risk of cancers were studied.

## Methods

Study Area: This cohort study was carried out in Isfahan, the third largest province of Iran (107,003 km^2^), which is located in the central part of Iran, between the latitudes of 30 and 34 degrees north of the equator, and longitude of 49–55 degrees, with altitude of 1590m. According to the last census which was performed by the Iranian Central Bureau of Statistics (ICBS) in Iran, the population of Isfahan was estimated around 4,815,863 (51% men and 49% women) and Isfahan was similar to other parts of country with respect to socioeconomic proportions, current demographic features, and lifestyles. Governmental, private and university hospitals, district health centers, and private physicians are the major places in Iran where health services are provided for people.

Registration: The registration of MS patients who are residents of Isfahan province was established in Isfahan Multiple Sclerosis Society (IMSS), the only referral system for Isfahanian MS patients. On April 5, 2003, all neurological wards in Isfahan hospitals mailed letters to patients who are diagnosed with MS by neurologists to IMSS registration system. In addition, MS cases were diagnosed by neurologist according to McDonald’s criteria registered to IMSS. Since IMSS is the only supporting center in the province where in providing insurance and health care facilities such as laboratory test, rehabilitation, and clinical treatments and drugs for MS patients are carried out, it is believed that almost all of them joined IMSS to receive this support. However, it should be considered that maybe there are few MS patients who believe that they do not need these facilities and refuse to register to this center leading to a small underestimation of Isfahan MS population. 

All patients who were registered in both IMSS registration database and cancer registration system have the same national ID number which helps an easy access in the identification of comorbidities in the same individual. In this study, we also utilized the database of Isfahan cancer registry wherein. The system tried to cover all cancer patients residing in Isfahan and in this regard, their population-based database has been completed since 2005. While MS patients in both registration systems have the same code identifying cancer and MS patients was performed manually since IMSS registration is not computerized. 

Diagnostic criteria: All MS patients were diagnosed by their neurologists according to the McDonald’s criteria which is approved international diagnostic criteria for MS (13). According to McDonald’s criteria, patients are classified to three groups: Definite MS, possible MS, and no MS that in our study being a case of definite MS was considered as an including criteria. Furthermore, diagnostic criteria for primary progressive multiple sclerosis (PPRMS) was used for the diagnosis of PPMS patients (14). While the rate of MS patient disability was assessed according to Kurtzke method which is also a common method for measuring expanded disability status scale (EDSS) in MS patients (15).

Statistical analysis: In the present study, all statistical analysis was performed by SPSS software (Version 22; SPSS Inc., Chicago, IL, USA). Also, in this cohort study, standardized incidence ratio (SIR), which was calculated by dividing the number of observed cases of cancer by the numbers of expected cancer cases, represented a measure of the relative risk of cancer. The number of expected cases of cancer was calculated by multiplying a stratified rate of cancer incidence in Isfahan by sum of age, sex and period-specific person years at risk in cohort. Wald’s test assuming a Poisson distribution of the observed cases was used for determining 95% confidence interval (CI) for SIR.

## Results

The MS cohort included 1718 MS patients (388 men and 1330 women) who were followed-up for average of 8 years, yielding a total of 13650 person-years at risk. Three types of MS were observed in MS population as: relapsing–remitting multiple sclerosis (RRMS): 1510 (87.89%); secondary progressive multiple sclerosis (SPMS): 110 (6.40%); primary progressive multiple sclerosis (PPMS): 98 (5.70%) and table 1 summarizes other sociodemographic characteristics and clinical features of studied patients. Overall, 23 cancer cases have been observed during 2006-2014 follow-up which approximately corresponds with 24 types of expected cancers [SIR=0.95 (95% CI, 0.8-1.10)]. 

**Table 1:** Sociodemographic characteristics and clinical features of studied patients

**Characteristics**	**Female N(%)**	**Male N(%)**
**Age at onset**		
<15 ≤15 and ≤50 ≥50(0.6)	70 (5.3) 1250 (94) 10 (0.7)	17 (4.3) 360 (92.7) 11 (3)
**Family history of MS**		
No Yes	1174 (88.3) 156 (11.7)	331 (85.3) 57 (14.7)
**Pattern of disease**		
Relapsing-remitting Primary progressive Secondary progressive	1180 (88.7) 70 (5.3) 80 (6)	328 (84.6) 30 (7.7) 30 (7.7)
**Occupation**		
Housewife/unemployed Student Employed	939 (70.6) 114 (8.6) 277 (20.8)	67 (17.2) 23 (6) 298 (76.8)
**Marital status**		
Single Married Divorced/Widowed	368 (27.7) 904 (68) 58 (4.3)	124 (32) 253 (65.2) 11 (2.8)
**Pregnancy history**		
Yes No	953 (71.7) 377 (28.3)	- -
Age(Mean±SD) (year)	31.8±9.2	33.7±9.6

The mean age of MS patients with cancer is 43.2±10.4 year. In history taking, it was revealed that only one patient had family history of cancer, while three others had history of MS in their family. Other clinical and paraclinical features of them are summarized in table 2. Among the 1718 MS patients with diagnosis of definite MS, 11 had breast cancer, 3 lymphoma, 3 nervous system cancer, and 6 of them had other types of cancer (table 3). Further analysis with respect to the cancer sites resulted in an increased risk of breast cancer [SIR=1.77 (95% CI, 1.12-2.76)], lymphoma [SIR=1.87 (95% CI, 1.64-2.20)] and cancers which are located in nervous system [SIR=2.30 (95% CI, 1.01-5.05)] and decreased risk of other cancers [SIR=0.40 (95% CI, 0.60-0.90] including: endocrine glands, bone, connective tissue, secondary and unspecified sites. While women with MS were at increased risk of cancers [SIR=1.09 (95% CI, 1.05-1.14), n=21)], men who developed cancers revealed a statistically significant decreased risk of cancers [SIR=0.39 (95% CI, 0.32-0.47), n=2)]. Number of observed and expected cancer cases and SIR with 95% CI among Isfahan MS patients are presented in table 3. 

**Table 2:** Clinical and paraclinical features of MS patients who developed cancers

**Characteristic**	**Female (%)**	**Male (%)**
Age (year)	44.1±9.9	34±4
Age at onset of MS (year)	31.6±8.7	25.5±3.64
EDSS* (year)	3.05±1.93	2.5±0.5
**Presenting symptom of MS**		
weakness cerebellar-brain stem sensory visual	38.1 14.3 4.7 42.9	50 0 0 50
**Family history of autoimmune disorders**		
Yes No	9.5 90.5	0 100

* Expanded Disability Status Scale

**Table 3:** Observed and expected number of cancers and standardized incidence ratios in a cohort of MS patients at anatomical sites.

**Cancer site**	**Observed**	**Expected**	**SIR**	**95%CI**
**All Cancers** Women Men	23 21 2	24.2 19.1 5.1	0.95 1.09 0.39	0.80-1.10 1.05-1.14 0.32-0.47
**Type of cancers **				
Breast Lymphoma Nervous system Others	11 3 3 6	6.2 1.6 1.3 14.9	1.77 1.87 2.30 0.4	1.12-2.76 1.64-2.20 1.01-5.06 0.60-0.90

SIR: Standard incidence ratio

## Discussion

Taken together, we found no increased risk of cancers among MS patients; however, men have a statistically significant decreased risk of cancers, while women showed a slight increases risk of cancers. Furthermore, our results revealed that the risk of lymphoma and nervous system cancer are considerably higher in MS patients, suggesting that the pathogenesis of these cancers and MS may be similar. The risk of breast cancer also showed a statistically significant increase. 

We found no evidence of an increased risk of other cancers after MS diagnosis. These results are similar to previous studies of this sort. Comorbidity of lymphoma and autoimmune and chronic inflammatory disorders was demonstrated by a population-based study in the Netherlands. Although this study did not address MS directly, it did study the co-occurrence of MS and other autoimmune inflammatory diseases such as rheumatoid arthritis, chronic inflammatory bowel disease, and ulcers, amongst others (16). The underlying pathways for these diseases are similar to those in MS. The link between autoimmune disorders and lymphatic cancer may lie in an imbalance between anti-inflammatory cytokines and pro-inflammatory cytokines (17).

It is the same imbalance that may also lead to reduce other cancer incidence in the MS population. Many other studies of which some of them had larger sample sizes, found an overall decreased rate of cancer as well. Because MS involves an upregulation of the immune system, to the degree of destroying the myelin sheath, protection against most cancers could be a beneficial side-effect. The increase in production of anti-tumor cytokines by helper T1 (Th1) cells, which are the main cells implicated in autoimmune inflammation, would help to prevent cancers from proliferating (18). 

As a consequence, this may be a viable link to the role that MS has in cancer immunity. Our study showed a significant increase outside of the 95% CI for the nervous system cancer. While the body produces anti-tumor cytokines in addition to attacking its own tissues, it is causing obvious inflammation in the CNS. Lesions of the brain are one of the diagnostic signs physicians use to decipher MS from other neurological diseases. Inflammation of CNS tissue is an obvious risk factor for primary cancers originating in the nervous system (19). Another possibility for the increase seen in nervous system cancer is the misclassification of benign MS lesions (12). Breast cancer and MS have been studied not only via epidemiological studies, but also through familial genomic studies as well. The BRCA1 gene mutation, caused by a frame shift, could be in close proximity to an MS gene, which would explain why many researchers have found more breast cancer cases in MS patients than expected (20). Of course, this was a single family and a single study, so the results may not be very significant. It is evidence that will need to be further examined as gene sequencing becomes more common. Another issue with this explanation is that BRCA mutations account for a very small percentage of breast cancer cases (21).

If MS patients who also have breast and ovarian cancers were to be tested for BRCA mutations, this could shed some light on the possibility of there being a significant MS gene. More likely than not, there are many genes that contribute to the likelihood of becoming afflicted with MS. The significant increase found for breast cancer is relatively common throughout these types of studies, and was reproduced here in our study. The other possible reason for these results could lie in women’s lifestyle choices, such as how long women wait to have children or if they have children at all. Another such study that found a statistically significant increase in breast cancer in their MS cohort was unable to account for the change with child-bearing related explanations (7).

One common treatment for MS is immunosuppressive (IS) therapy. A study looking at IS treatments and cancer found that the patients who were treated with IS had three times greater risk of developing cancer than an MS patient who never received IS (22). This study found a particular trend towards breast cancer, cancer in the urinary tract, digestive tract, and skin. While the overall risk of cancer was reduced for the portion of the cohort without any history of IS treatment, including a reduced risk for breast cancer in women, the story was different in the treatment positive portion of the cohort. The chances of getting these cancer types increased between the two groups, but did not exceed significance with regard to the general population. 

There is a possibility that any increase in cancer prevalence in an MS cohort is due to increased surveillance by the patient. Breast cancer in particular is a special case where patient concern may lead to overdiagnosis. Women who experienced breast cancer in situ may have never been diagnosed if yearly screening tools were not used such as mammography and breast exam (23). The average MS patient will spend more time visiting doctors than an unaffected individual, which means there is a tendency towards over-vigilance. This attention to health may explain the discrepancies between the general population and MS patients when it comes to breast cancer. When MS patients were followed-up as well as their parents, no genetic link was found between patients who have cancer and MS comorbidity and cancer in their parents (24). 

This suggests that there is no significant genetic link between cancer and MS. There was an observable increased risk of urinary tract cancer and brain tumors in their MS cohort, but an overall decreased risk of cancer, can be attributed to changes in behavior and immunological properties of the disease.

In conclusion, our results further support the hypothesis that MS significantly affects only certain cancers, either in a protective or associative manner. While we found no significant change in cancer rates overall from the general population, we did find men to be less likely to develop cancer after an MS diagnosis. Women had an opposite response. 

The rates of cancer incidence for women were above the 95% confidence interval calculated from general population rates. Further statistical analysis shows that the rates of breast cancer, cancers located in the nervous system, and lymphomas increased in the cohort of our 1718 patients. This could be due to many factors, including inflammatory consequences of autoimmune disease, patient behavior, genetic predispositions, and intensified health surveillance after diagnosis. The rates of all other cancers reduced in our cohort, suggesting that upregulated immune function may provide protective effects to MS patients against cancer. More research studies in this area are needed via epidemiology and experimental study designs that will be necessary to fully understand if there is a link between certain types of cancer and MS, and if so, what is that link.
